# Supplementary material for: Clonal dynamics in osteosarcoma defined by RGB marking
Source: Nat Commun. 2018 Sep 28;9:3994. doi: 10.1038/s41467-018-06401-z (PMC6162235; doi:10.1038/s41467-018-06401-z)
Supplement: Supplementary file 1 — Supplementary Information [file 41467_2018_6401_MOESM1_ESM.pdf]

Supplementary Information

Clonal dynamics in osteosarcoma defined by RGB marking

Gambera *et al.*

CONTENTS:

- Supplementary Figure 1 Validation of RGB marking in RAINBONE cells.
- Supplementary Figure 2 RGB marking of RAINBONE cells is stable during *in vitro* culture.
- Supplementary Figure 3 ViSNE analysis of RAINBONE cells over time.
- Supplementary Figure 4 MOI estimation and RAINBONE cell marking.
- Supplementary Figure 5 Increasing clonal complexity reduces tumour growth.
- Supplementary Figure 6 Clonal organization of primary tumours and metastatic lung nodules.
- Supplementary Figure 7 Insertion site analysis (LAM-PCR).
- Supplementary Figure 8 *In vitro* and *in vivo* chromosomal instability and karyotype variability.
- Supplementary Figure 9 Heterogeneity between metastatic and primary tumour clones.
- Supplementary Figure 10 Analysis of dominant clone decolouring.
- Supplementary Figure 11 Dominant clones maintain their polyclonality during *in vivo* passaging.
- Supplementary Figure 12 Uncropped electrophoretic gels.
- Supplementary Table 1 Chromosomes analysis using spectral karyotyping (SKY).
- Supplementary Table 2 RGB lentiviral vectors insertion sites analysis.
- Supplementary Table 3 Ad-Cre vector efficiency estimation.

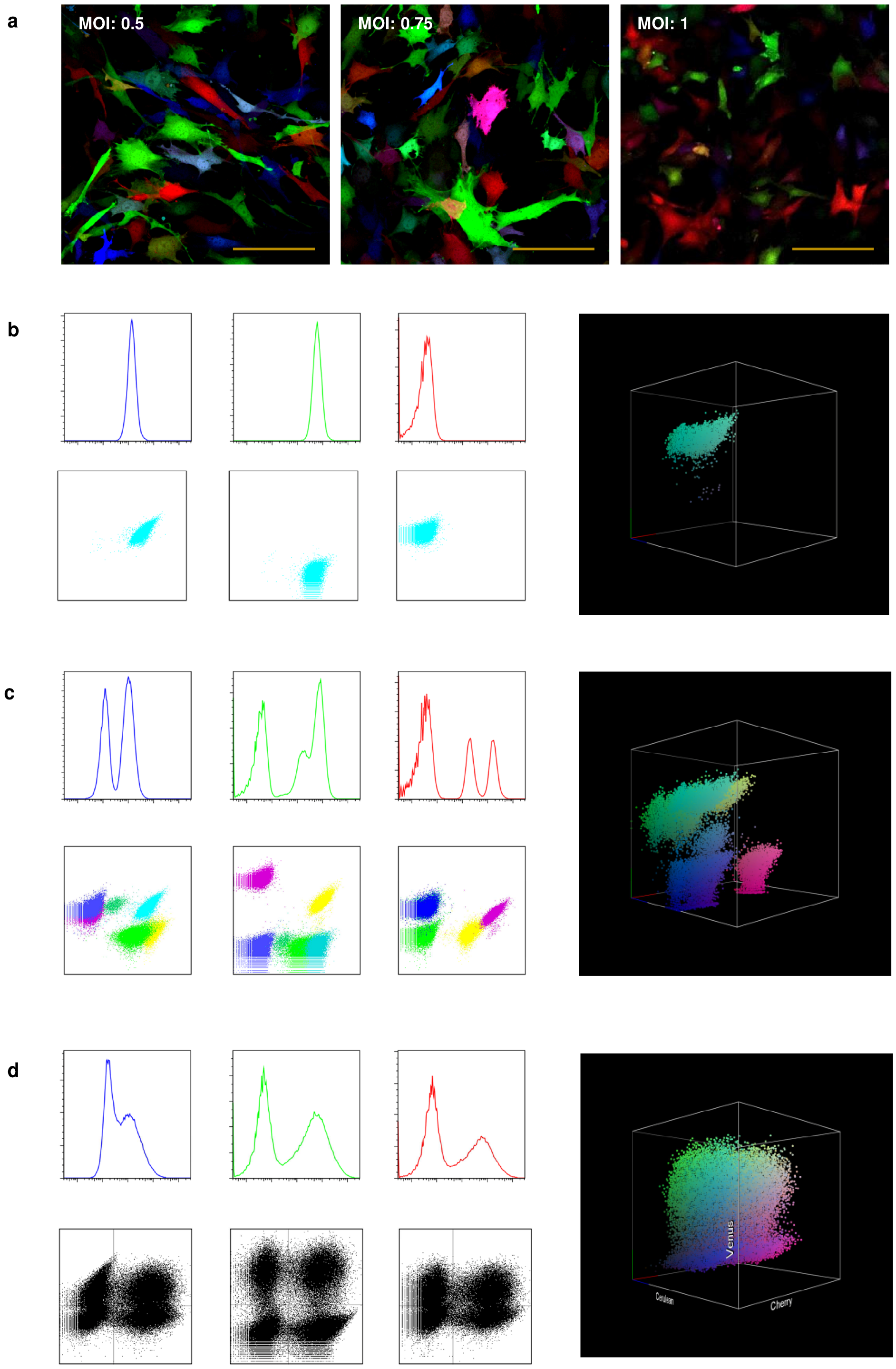

Supplementary Figure 1 Validation of RGB marking in RAINBONE cells.

a, Representative images of confocal microscopy showing multicolour RAINBONE cells generated after lentiviral transduction using different equimolar MOIs of RGB vectors (ratio 1:1:1). **b,** Representative fluorescence distribution per colour of monoclonal cell lines; obtained by limiting dilution. **c,** Representative fluorescence distribution of oligoclonal populations generated by an *in vitro* mixture of unicolour clones. **d**, Representative fluorescence distribution of polyclonal RAINBONE cells. Different clonal populations are

indicated in black in dot-plots. Histograms: from left to right, single fluorescence distribution of Cerulean (blue), Venus (green), and mCherry (red). Dot plots: from left to right, fluorescence distribution of Venus vs. Cerulean; Venus vs. Cherry; Cherry vs. Cerulean, according to the X vs. Y order. 3D plots: cube visualization of the Cerulean, Venus, and Cherry fluorescence distribution according to the X vs. Y vs. Z convention. Dots are coloured according to the fluorescent marker combination and intensity. Orange bars = 100 µm.

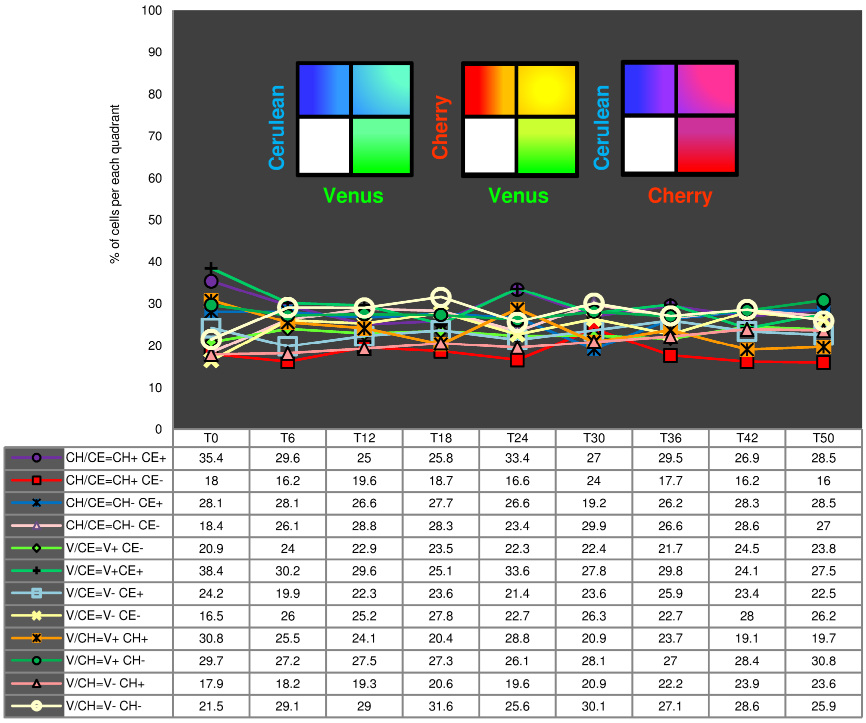

	T0	T6	T12	T18	T24	T30	T36	T42	T50
CH/CE=CH+ CE+	35.4	29.6	25	25.8	33.4	27	29.5	26.9	28.5
CH/CE=CH+ CE-	18	16.2	19.6	18.7	16.6	24	17.7	16.2	16
CH/CE=CH- CE+	28.1	28.1	26.6	27.7	26.6	19.2	26.2	28.3	28.5
CH/CE=CH- CE-	18.4	26.1	28.8	28.3	23.4	29.9	26.6	28.6	27
V/CE=V+ CE-	20.9	24	22.9	23.5	22.3	22.4	21.7	24.5	23.8
V/CE=V+CE+	38.4	30.2	29.6	25.1	33.6	27.8	29.8	24.1	27.5
V/CE=V- CE+	24.2	19.9	22.3	23.6	21.4	23.6	25.9	23.4	22.5
V/CE=V- CE-	16.5	26	25.2	27.8	22.7	26.3	22.7	28	26.2
V/CH=V+ CH+	30.8	25.5	24.1	20.4	28.8	20.9	23.7	19.1	19.7
V/CH=V+ CH-	29.7	27.2	27.5	27.3	26.1	28.1	27	28.4	30.8
V/CH=V- CH+	17.9	18.2	19.3	20.6	19.6	20.9	22.2	23.9	23.6
V/CH=V- CH-	21.5	29.1	29	31.6	25.6	30.1	27.1	28.6	25.9

Supplementary Figure 2 **RGB marking of RAINBONE cells is stable during *in vitro* culture**.

Quantification of the fluorescence distribution during 50 days of *in vitro* culture of RAINBONE cells. Each coloured line indicates the quantification value of single positive, double positive, or double negative populations in each flow cytometry dot plot combination (Venus vs. Cerulean; Venus vs. Cherry; Cherry vs. Cerulean according to X vs. Y order). Statistical analysis indicated no significant correlation between days of culture and population frequency.

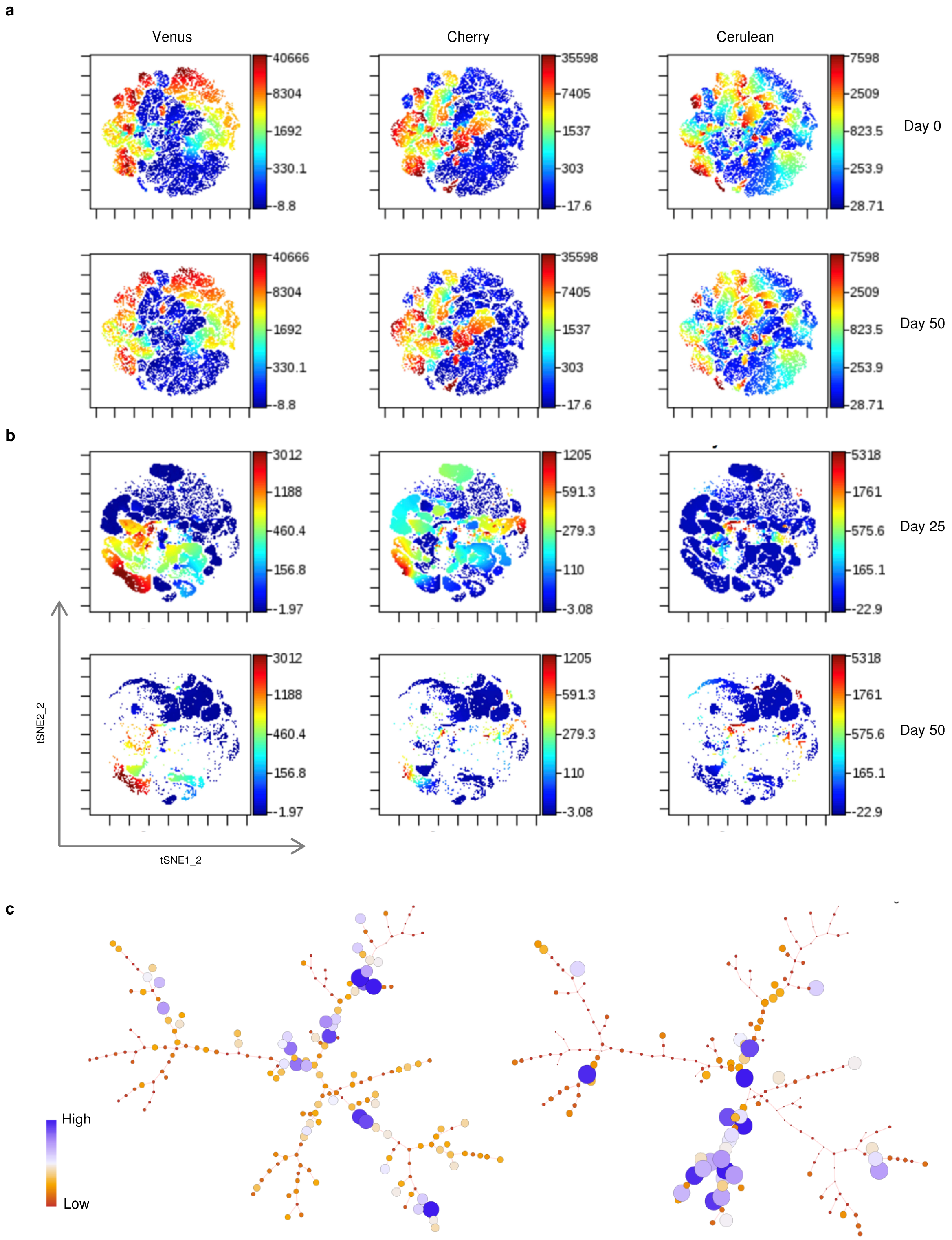

Supplementary Figure 3 **ViSNE analysis of RAINBONE cells over time.**

ViSNE maps showing clonal populations of RAINBONE cells *in vitro* **(a)** and *in vivo* in ectopic osteoinductive implants **(b)**. Each plot represents the 2D scattered visualization of the cell population per fluorescent colour according to the tSNE1 and tSNE2 relatedness. Colours are according to the heat map. **c,**

SPADE tree clonal composition of RAINBONE tumours after 25 days (left) and 50 days (right). In this case, colours and size represent the population frequency.

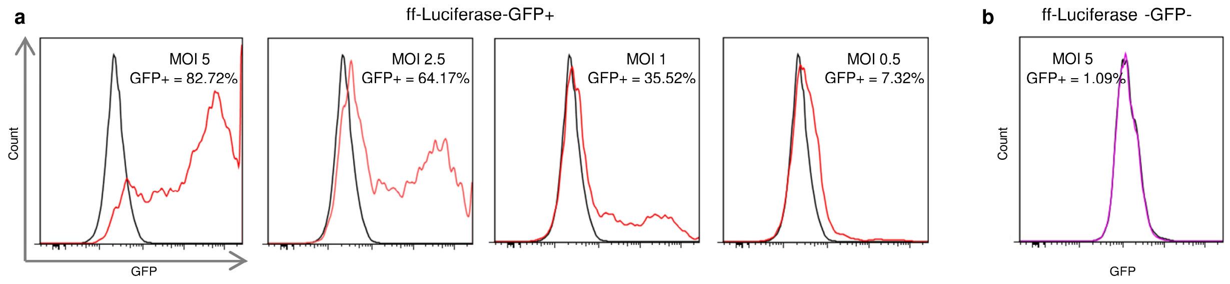

Supplementary Figure 4 **MOI estimation and RAINBONE cell marking.**

p53$^{-/-}$*Rb*$^{-/-}$ BM-MPCs were transduced with ff-Luciferase-GFP+ and ff-Luciferase-GFP- lentiviral vectors. **a,** Histograms showing MOI-dependent GFP expression of ff-Luciferase-GFP-transduced *p53*$^{-/-}$*Rb*$^{-/-}$BM-MPCs. Non-transduced cells were used as controls (black) **b,** Histogram showing *p53*$^{-/-}$*Rb*$^{-/-}$ BM-MPCs transduced with ff-Luciferase vector lacking the GFP reporter. Non-transduced *p53*$^{-/-}$*Rb*$^{-/-}$ BM-MPCs were used as negative controls (black).

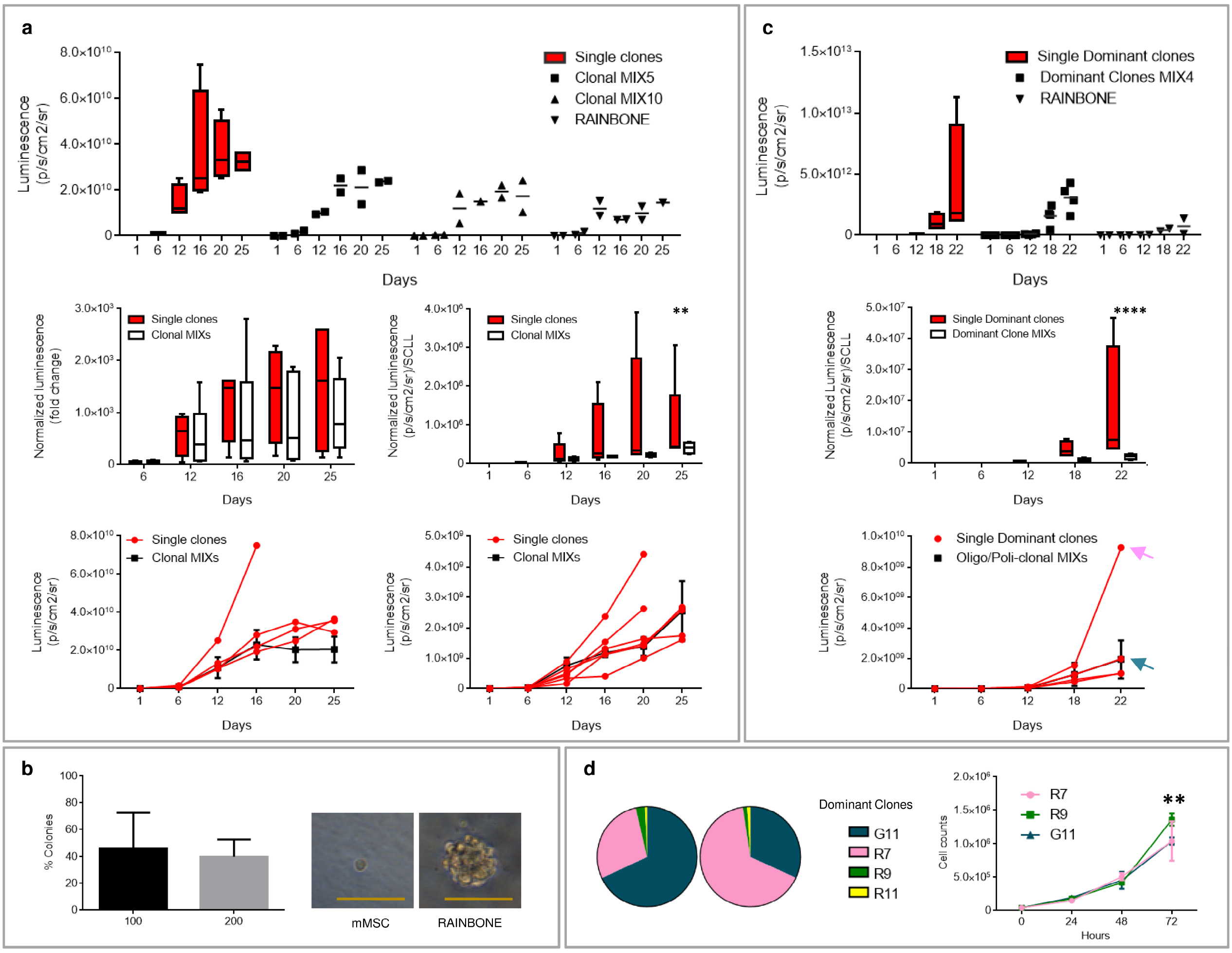

Supplementary Figure 5 Increasing clonal complexity reduces tumour growth.

a, Bioluminescent *in vivo* growth quantification of tumours generated by monoclonal (single clones; n=10), oligoclonal (clonal MIX5, n=6; clonal MIX10; n=2), and polyclonal (RAINBONE cells; n=6) populations during 25 days of *in vivo* growth (two different experiments, total n=24). Mono- and oligo-clonal populations were obtained by mixing 5 (clonal MIX5) or 10 (clonal MIX10) clones obtained from *in vitro* limiting dilution of RAINBONE cells. The upper graph shows representative luciferase activity. Graphs in the middle, show normalized luciferase activity of each individual experiment. In the lower part, tumour growth kinetic of different single clones versus clonal MIXs is represented, two different experiments. The panel show a tendency of slower tumour growth when increasing clonal complexity and a heterogeneous tumour growth kinetics of each monoclonal population. **b,** RAINBONE cells soft-agar colony formation assay (n=8). Two different seeding density were tested (100 or 200 cells/per well); error bars represent standard deviation. In the right part, representative images of RAINBONE colonies versus wild-type murine MSCs (orange

bars= 100 μm). **c,** Bioluminescent *in vivo* tumour growth quantification generated by monoclonal, oligoclonal, and polyclonal populations during 25 days of secondary transplantation (n=10). Four monoclonal cell lines were derived by FACS-sorting of dominant clones isolated from primary tumours; oligoclonal mix represents a population obtained by mixing the previous 4 dominant clones (clonal MIX4; n=4); RAINBONE cells were used as control (n=2). The upper graph shows luminescence quantification of the three experimental groups. Graphs in the middle, show normalized luciferase activity. In the lower part, is represented the growth kinetics for tumours generated by monoclonal populations. Arrows indicated G11 and R7 clones. **d,** the left panel shows the composition of 25 days-secondary tumours of two ClonalMIX4 (G11, R7, R9, and R1) populations, analysed by flow cytometry. The right panel shows *in vitro* growth assays for individual clones (n=3). All data are presented as single values or as means and standard deviations; for box and whiskers plots, means plus minimum and maximum values are presented; statistic tests: two-tail unpaired t-Test and two-way ANOVA, Bonferroni post-hoc test, CI: 95%, alpha: 0.05. P<0.05 (*), P<0.01 (**), P<0.001 (***), P<0.0001 (****).

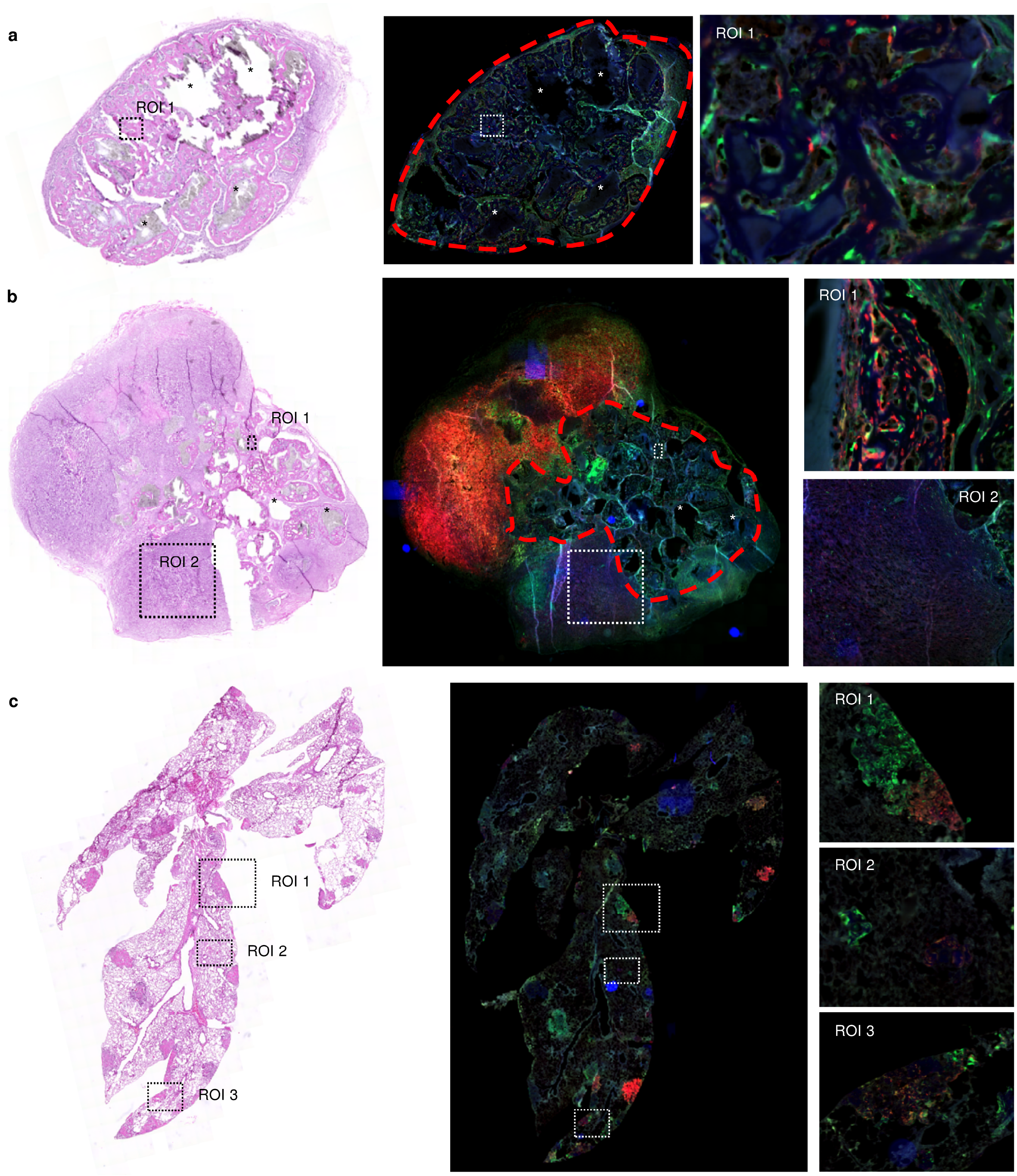

Supplementary Figure 6 **Clonal organization of primary tumours and metastatic lung nodules**.

a-b, Virtual microscopy reconstruction map of subcutaneous tumours extracted after 25 days (**a**) and 50 days (**b**). From left to right, each panel includes Hematoxylin/Eosin staining, the fluorescent map reconstruction of the whole tumour, and a detailed view of a heterogeneous and monoclonal area. **c,** Virtual reconstruction of entire lung lobules and metastatic nodules. From left to right, Hematoxylin/Eosin staining, macroscopic reconstruction of the whole lobules, and the detailed view of monoclonal and oligoclonal metastatic nodules.

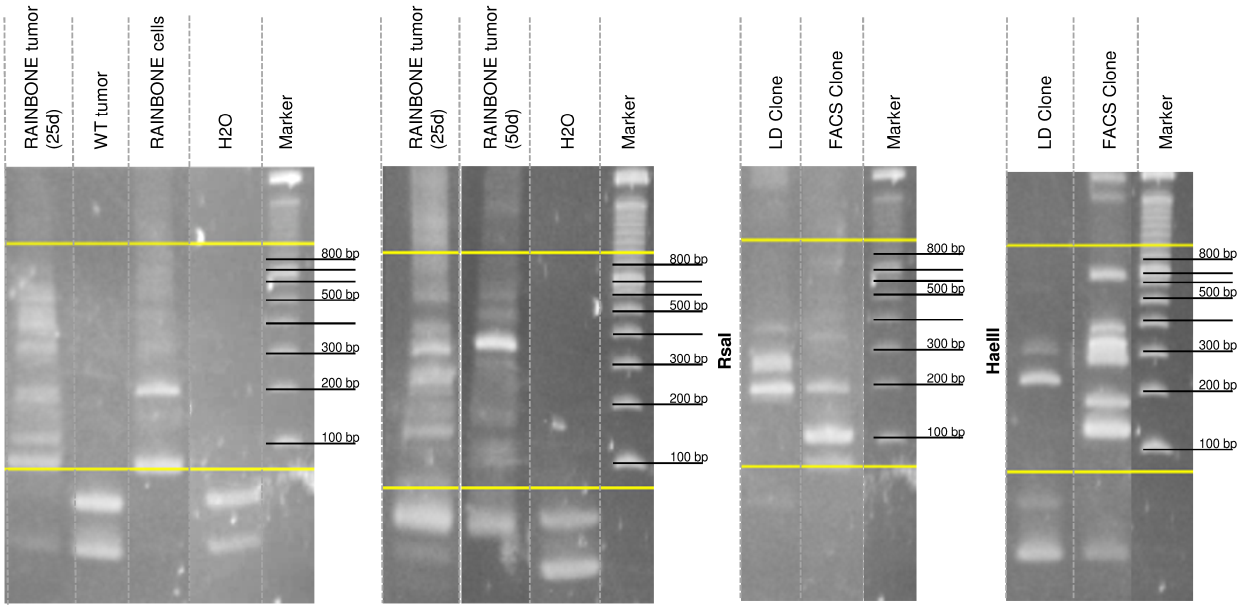

<u>Supplementary Figure 7</u> **Insertion site analysis (LAM-PCR).**

LAM-PCR analysis showing the amplification of different provirus integration sites in RAINBONE tumours. From left to right: enlarged pictures from Fig. 2f, Fig. 3c, and Fig. 3g. Vertical lines were added where non-informative lanes were excised. Horizontal yellow lines demarcate the standard size range of the PCR products to exclude amplification artefacts. Negative control = water; WT tumour = tumour generated by unmarked cells.

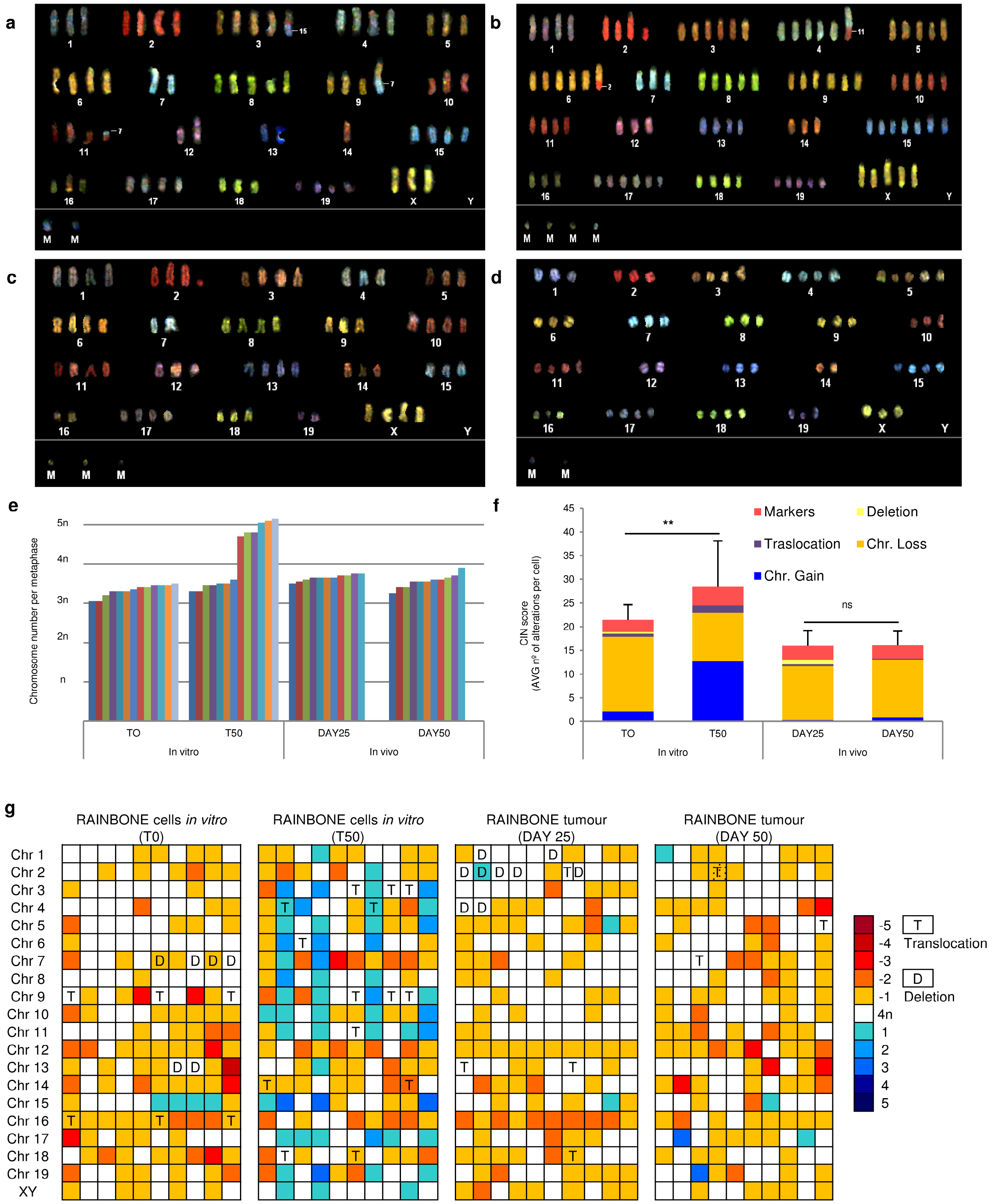

Supplementary Figure 8 ***In vitro*** **and** ***in vivo*** **chromosomal instability and karyotype variability.**

Representative SKYs of RAINBONE cells at day 0 **(a)** and day 50 **(b)** of *in vitro* culture. Representative SKY karyotypes of RAINBONE tumours at day 25 **(c)** and day 50 **(d)**. **e-f,** Chromosome number

quantification **(e)** and chromosomal instability score **(f)** of *in vitro* RAINBONE cells and *in vivo* RAINBONE tumours. Error bars represent the standard deviation. Statistical testing was according to one-way ANOVA: $p<0.0001$, Bonferroni post hoc test, CI: 95%, alpha: 0.05, $^{**}p<0.01$. **g,** Heatmap of 10 representative metaphases showing the high genomic heterogeneity detected in the different experimental points.

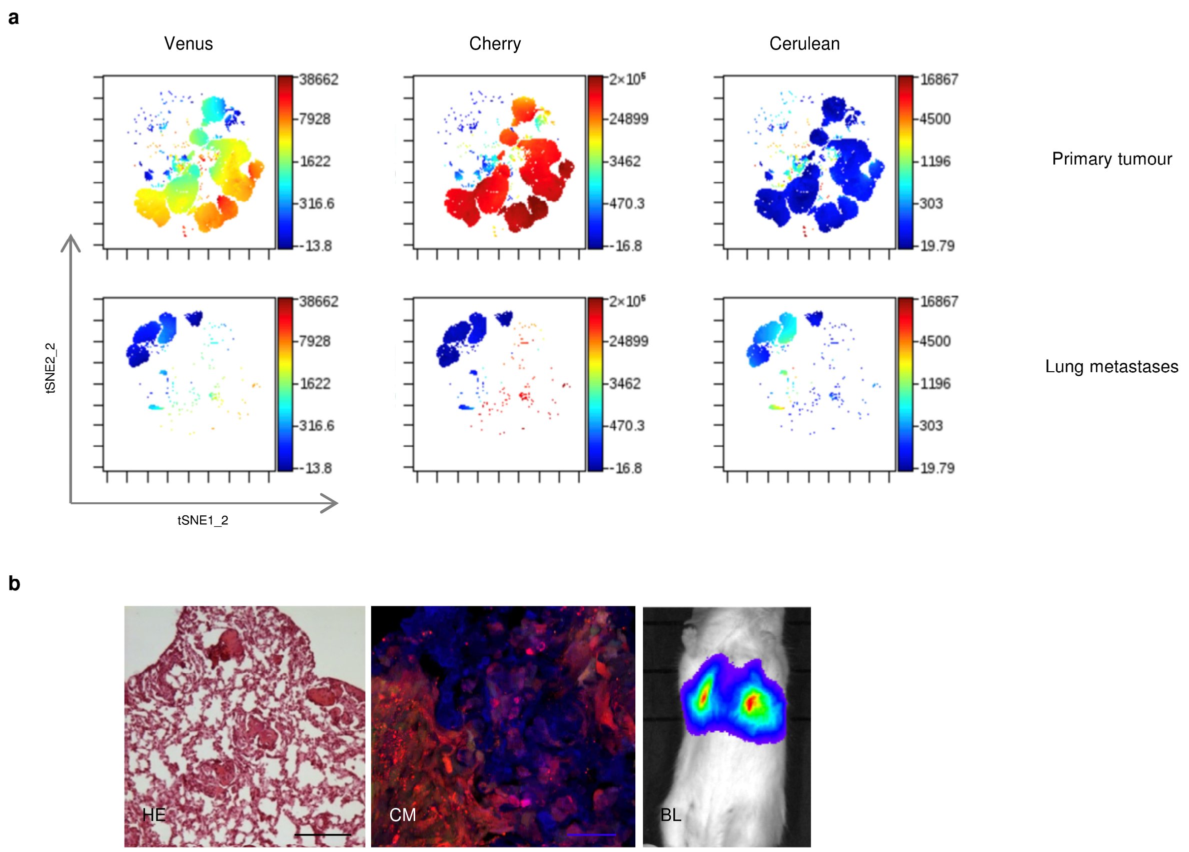

Supplementary Figure 9 **Heterogeneity between metastatic and primary tumour clones.**

a, ViSNE maps showing clonal populations developing in RAINBONE primary tumours and metastases. Each plot represents the 2D scattered multidimensional visualization based on the tSNE population relatedness. Colours are according to the heat map. **b,** *In vivo* assay of two independent sorted FACS clones tested for metastatic potential (n=4). From left to right, intravenously induced lung metastases detected by classical Hematoxylin/Eosin staining (HE), confocal microscopy (CM), and bioluminescent study (BL) at final time point. Black bar = 200 µm; Blue bar = 50 µm.

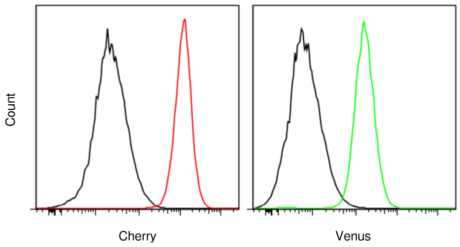

Supplementary Figure 10 **Analysis of dominant clone decolouring.**

Histograms represent the fluorescent signal distribution of a representative clone before (colored) and after (black) Ad-cre transduction. Ad-cre recombination efficiency for three different FACS clones was estimated as the percentages of positive cells for red (Cherry), green (Venus), and Cerulean (Blue) fluorescent markers, before and after Ad-Cre transduction. Results are presented in Supplementary Table 2.

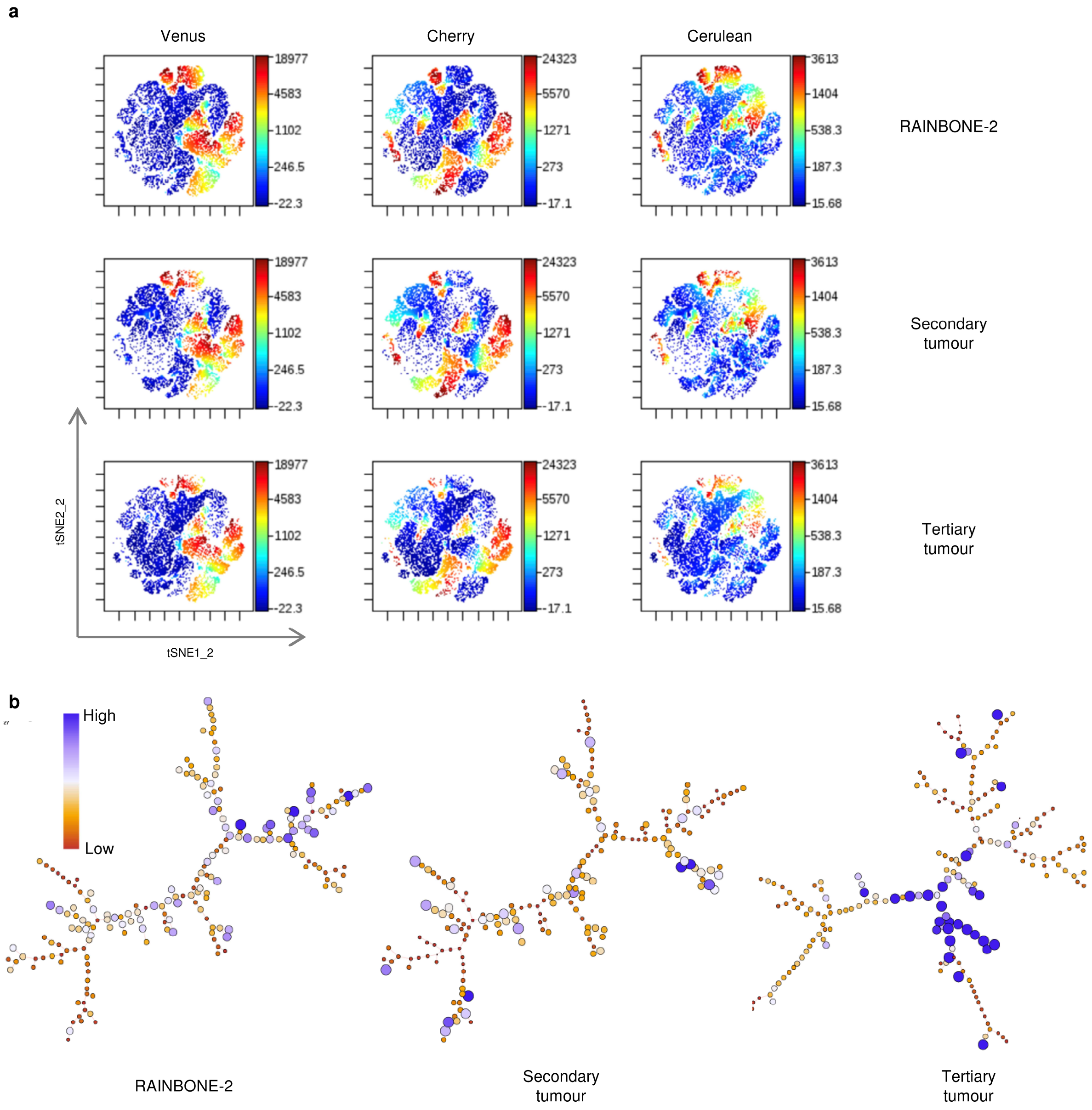

Supplementary Figure 11 **Dominant clones maintain their polyclonality during *in vivo* passaging.**

a, ViSNE multidimensional visualization of recoloured tumour FACS clones *in vitro* (RAINBONE-2) and after serial ectopic osteoinductive tumour generation. Each plot represents a heat map visualization of studied populations per fluorescent colour. **b,** SPADE tree population reconstruction of RAINBONE-2 cells (left) and secondary (middle) and tertiary (right) tumours; the dot size and colour represent the population frequency.

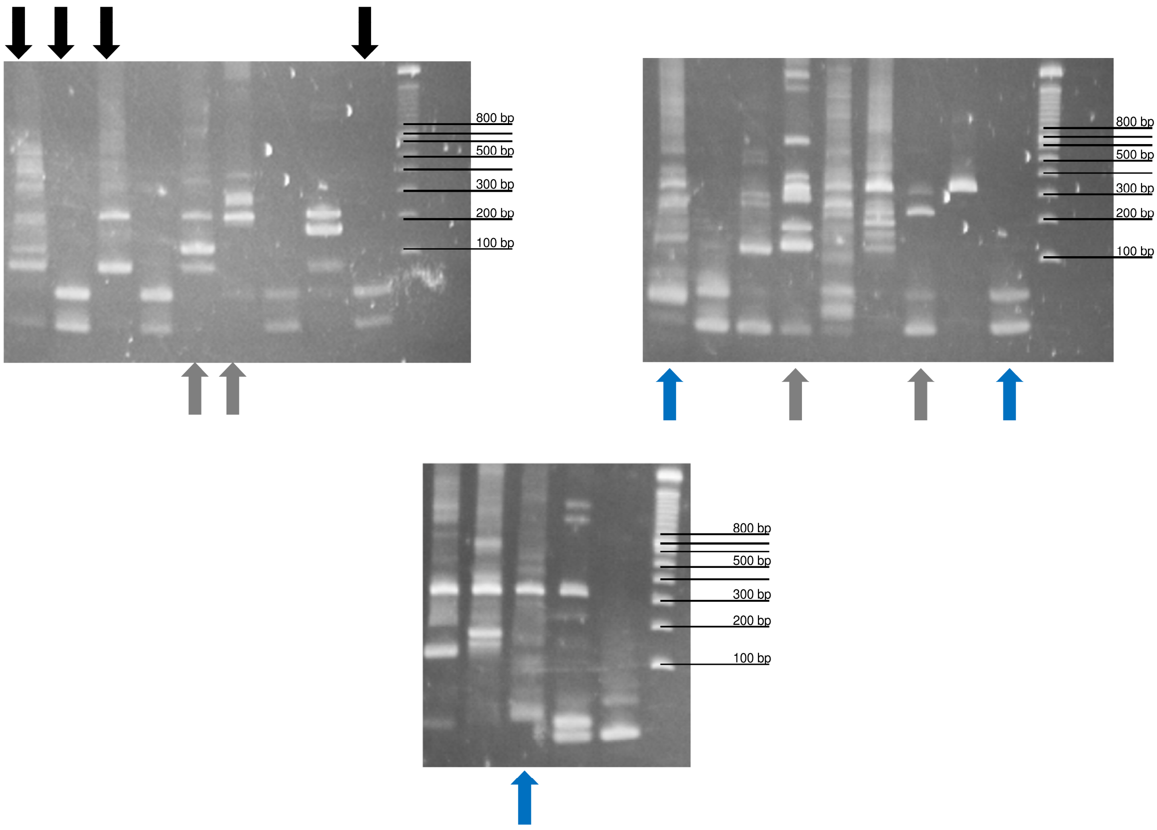

<u>Supplementary Figure 12</u> **Uncropped electrophoretic gels.**

The figure shows uncropped images of polyacrylamide gels employed to separate LAM-PCR products. Arrows indicates lanes included in Fig. 2f (black arrows), Fig. 3c (blue arrows) and Fig. 3g (grey arrows).

SUPPLEMENTARY TABLES:

Supplementary Table 1 Chromosomes analysis using spectral karyotyping (SKY).

**Composite karyotype formula:**
RAINBONE cells *in vitro* (T0)
66-76,-X,-2,der(4)t(4;11),-5,-7,der(9)t(7;9),-10,-11,-12,-13,-14x2,-15,der(16)t(3;16;19),-16x2,-17,-18,1-7mar[cp10]
RAINBONE cells *in vitro* (T50)
66-103,+X,-1,-2,+3,+4,+der(4)t(4;11),+6,-7x2,+9,der(9)t(7;9),+11,-12,-13,-14,+15,der(16)t(3;16;19),-16,+17,-18,+19,1-7mar[cp14]
RAINBONE tumour *in vivo* (T25)
70-75,-X,del(2),-4,-5,-7,-12,-14,-16x2,-17,-18,-19,2-4mar[cp11]
RAINBONE tumour *in vivo* (T50)
65-78,-X,-1,-2,-4,-6,-7,-8,-9,-10,-11,-12,-13,-14,-16,-17,-18,-19,2-4mar[cp10]

Supplementary Table 2 RGB lentiviral vectors insertion sites analysis.

Specimen	Restriction Enzyme	Chrom Location	Sequence
LD clone	Rsal	Too short	ACACCAG
	Rsal	Chrom 10: 129116926	ACTAAAATCATTTCAGCACTTTTTGA AAGAGATGCAGCATCTAAACTCTTCT TGAGCCTAAACATTCATTCTAAACAT GAGTAAGATCAGAGTATCATAAATAT ATTTTAAAACTCAATAATTAGAGACT CTATAAAGAAAAAAAACTGAATCT
FACS clone	Rsal	No match	ACAAGCAAAAAGCAGATCTTGTCTTC ATTGGGAGTGAATTAGCCCTTCCAGT
	Rsal	No match	GGGTTCCCTAGTTAGCCAGAGAGCTC CCAGGCTCAGATCTGGTCTAACCAGA GAGACCCAGT
FACS clone	HaeIII	Chrom 9: 113826325	AACCAAGCACTACTTGACTGGAATTA AGG
	HaeIII	Chrom 6: 141492663	TACCATATGGATCAATCGTTGGCTTTT TTTCGTGG
	HaeIII	Chrom 9: 113826325	CCTTAATTCCAGTCAAGTAGTGCTTG GTT

LD clone: clone obtained by limiting dilution, FACS clone: clone obtained by FACS sorting, Chrom: Chromosome.

Supplementary Table 3 Ad-Cre vector efficiency estimation.

Sample name	% Cherry	% Venus	% Cerulean
FACS Clone 1	99.7%	98.4%	0.6%
Ad-Cre FACS Clone 1	1.4%	2.0%	0.0%
FACS Clone 2	0.0%	89.6%	72.0%
Ad-Cre FACS Clone 2	0.1%	0.0%	2.7%
FACS Clone 3	98.3%	0.1%	97.7%
Ad-Cre FACS Clone 3	0.3%	0.0%	1.6%

FACS clone: clone obtained by FACS sorting, %: percentage of cells expressing RGB markers in flow cytometry analysis.
