# Supplementary material for: Clonal dynamics in osteosarcoma defined by RGB marking
Source: Nat Commun. 2018 Sep 28;9:3994. doi: 10.1038/s41467-018-06401-z (PMC6162235; doi:10.1038/s41467-018-06401-z)
Supplement: Supplementary file 3 — Description of Additional Supplementary Files [file 41467_2018_6401_MOESM3_ESM.pdf]

Description of Additional Supplementary Files

File Name: Supplementary Movie 1
Description: SPADE-tree clonal composition of RAINBONE cells in vitro; colour represents population frequency.
